# A concise guide of contemporary cardiovascular imaging practices to differentiate athlete’s heart in the gray zone

**DOI:** 10.1007/s10741-025-10541-y

**Published:** 2025-06-27

**Authors:** Efstathios D. Pagourelias, Styliani Ouzouni, Panagiotis Salmatzidis, Theocharis Sargiannidis, Eleni Tsiouli, Dimitrios Ntelios, Evangelia Kouidi, Vasileios P. Vassilikos

**Affiliations:** 1 Third Cardiology Department, Medical School, Cardiomyopathy UnitHippokrateion University HospitalAristotle University of Thessaloniki, Thessaloniki, Greece; 2 Sports Medicine Laboratory, Department of Physical Education and Sports Science, Aristotle University of Thessaloniki, Thessaloniki, Greece

**Keywords:** Athlete’s heart, Cardiomyopathies, Advanced echocardiography, Cardiovascular magnetic resonance, Diagnostic algorithm

## Abstract

Athlete’s heart comprises various structural and functional adaptations, imposed by systematic training and intended to serve the increased needs of the body during exercise. In most cases, athletic cardiac remodeling presents mild characteristics that are easily distinguishable from pathologic entities. However, common inherited cardiomyopathies such as hypertrophic, dilated, or arrhythmogenic may also affect athletes or athletic individuals, while athlete’s heart in a more pronounced form (frequently called “gray” zone) should be distinguished from early stages of the above-mentioned cardiomyopathies. Based on these assumptions, cardiovascular imaging remains the key process that should be applied to accurately differentiate between normal and abnormal phenotypes, facilitating thus pre-participation screening along with early detection and handling of underlying cardiomyopathies. Recent advances in both echocardiography and cardiovascular magnetic resonance offer new diagnostic potentials, making, however, “method” and “time” selection rather complicated. The aim of this review is to provide a short and comprehensive guide for differentiating athlete’s heart in the gray zone from cardiomyopathies, encompassing all contemporary tools of imaging modalities into easily applicable and hierarchically appropriate algorithms.

## Introduction—definition of athlete’s heart

Athlete’s heart (AH) is the set of structural and functional adaptations of myocardium to systematic exercise [1]. These changes allow the heart to respond to its increased task of delivering blood and oxygen to the tissues. The basic prerequisite for AH’s adaptations is systematic training for 3 to 4 h per week over 3 months [2]. Exercise leads to hemodynamic changes (higher pulmonary oxygen uptake, increased heart rate and preload) which result in Exercise-Induced Cardiac Remodeling [2].

Morpho-functional alterations of AH are imposed by multiple factors [3–5]. The type of training protocols and exercise intensity/training volume influence the variability of left ventricular dimensions and mass (at about 50%), as well as the right ventricular (RV) size. As the “static” component increases, a concentric remodeling of the left ventricle (LV) is observed. As the “dynamic” component increases, an eccentric remodeling of the LV prevails [2]. Based on Mitchel’s classification, in athletes with high dynamic and low static demands (e.g., runners), eccentric LV hypertrophy is observed. In athletes with high static demands (e.g., weightlifting), the LV is characterized by concentric hypertrophy accompanied by a reduction in its dimensions [5, 6]. In sports with high dynamic and static demands (e.g., cycling), there is both hypertrophy and dilation of the LV [5, 6], as well as RV dilation [2]. Gender also plays an important role, with female athletes showing similar but smaller changes compared to their male fellow athletes. A third factor is race, with black athletes having more pronounced changes in cardiac structure and functional/electrocardiographic characteristics compared to white athletes. Twelve percent of black athletes present a wall thickness greater than 12 mm [4], with only 2% of white athletes presenting such a finding [3, 5].

In most cases, morphological alterations in AH are within “normal” range, or they are accompanied by “supra-normal” functional adaptations. On the other hand, less than 2% of highly trained athletes exhibit hypertrophy or dilatation magnitude of LV and or RV that exceed the upper normal range, overimposing the range of various cardiomyopathy types, such as hypertrophic, dilated, and/or arrhythmogenic [3, 6]. In these cases, early and accurate differential diagnosis is essential to prevent life-threatening events during exercise [6] and guide prevention strategies and therapeutic regimes.

Rather than detailing a wide comparison between studies on cardiomyopathies and AH and for the shake of simplicity and clinical applicability, we suggest the development of a concise guide that shows the differences between the above mentioned entities and encompasses all contemporary tools of imaging modalities into an easy to use, helpful tool for clinicians.

### Hypertrophic cardiomyopathy (HCM)

#### Definition of gray zone

When LV wall thickness ranges between the diagnostic limit for HCM and the maximal value of hypertrophy observed in AH, the challenge begins [7]. This reference range, frequently mentioned as a “gray zone,” is often different between the two genders, with LV wall thickness between 13–15 mm in men and 12–13 mm in women to be considered as “normal” [5–7]. Despite that, any athlete with LV wall thickness > 12 mm should be considered as potential HCM patient [5–7].

#### Classical echocardiography

Two-dimensional echocardiography is the one that initially poses the dilemma of differential diagnosis of HCM from AH through the determination of the maximum LV wall thickness. Beyond this, the type of hypertrophy (eccentric in the HCM vs concentric in the athletic heart) may also be a useful element in the differential diagnosis of the two entities, by also detecting early phenotypic features of the disease. For example, the presence of myocardial crypts, the appearance of elongated mitral valve leaflets, and/or systolic anterior motion (SAM) of the anterior leaflet of the mitral valve may favor the diagnosis of HCM [6–8]. Additionally, assessing the end-diastolic diameter of the LV or the size of the left atrium can help in differential diagnosis, since the AH is accompanied by larger dimensions and improved functionality of the atrium [6–8]. Apart from the two-dimensional imaging, Doppler echocardiography can also help. A more advanced stage of diastolic dysfunction and/or impaired velocity values of tissue-Doppler are hallmarks of HCM. However, their occurrence accompanies more advanced stages of the disease and not mild or incipient forms of HCM, which mainly need to be distinguished from AH [9, 10]. Finally, the combination of the above morphological and functional markers in one score [9, 10] (Table 1) may better contribute to the differential diagnosis, since the recruitment of more indices better distinguishes these two conditions.

**Table 1:** Diagnostic score for discriminating mild hypertrophic cardiomyopathy from adaptive left ventricular hypertrophy (athlete’s heart). A score value > 5 has been proposed as a distinguisher of HCM [REF 9]

Variable	Cut off limit	Score points
LVEDD	≤ 4.74 cm	**2**
MITRAL IVRT	> 94 ms	**2**
SEPTUM Em	≤ 9.5 cm/sec	**2**
MITRAL DT	> 200 ms	**1**
TRIC E/A	≤ 1.63	**1**
RWT	> 0.445	**1**
BNP REST	> 9.84 pg/mL	**1**

*LVEDD* left ventricular end-diastolic diameter, *MITRAL IVRT* mitral inflow isovolumic relaxation time, *SEPTUM Em* Em wave obtained by Tissue Doppler at the septal corner of mitral annulus, *MITRAL DT* mitral inflow deceleration time, *TRIC E/A* tricuspid inflow E/A, *RWT* relative wall thickness, *BNP Rest* resting BNP values

#### Strain echocardiography

**Fig. 1:**
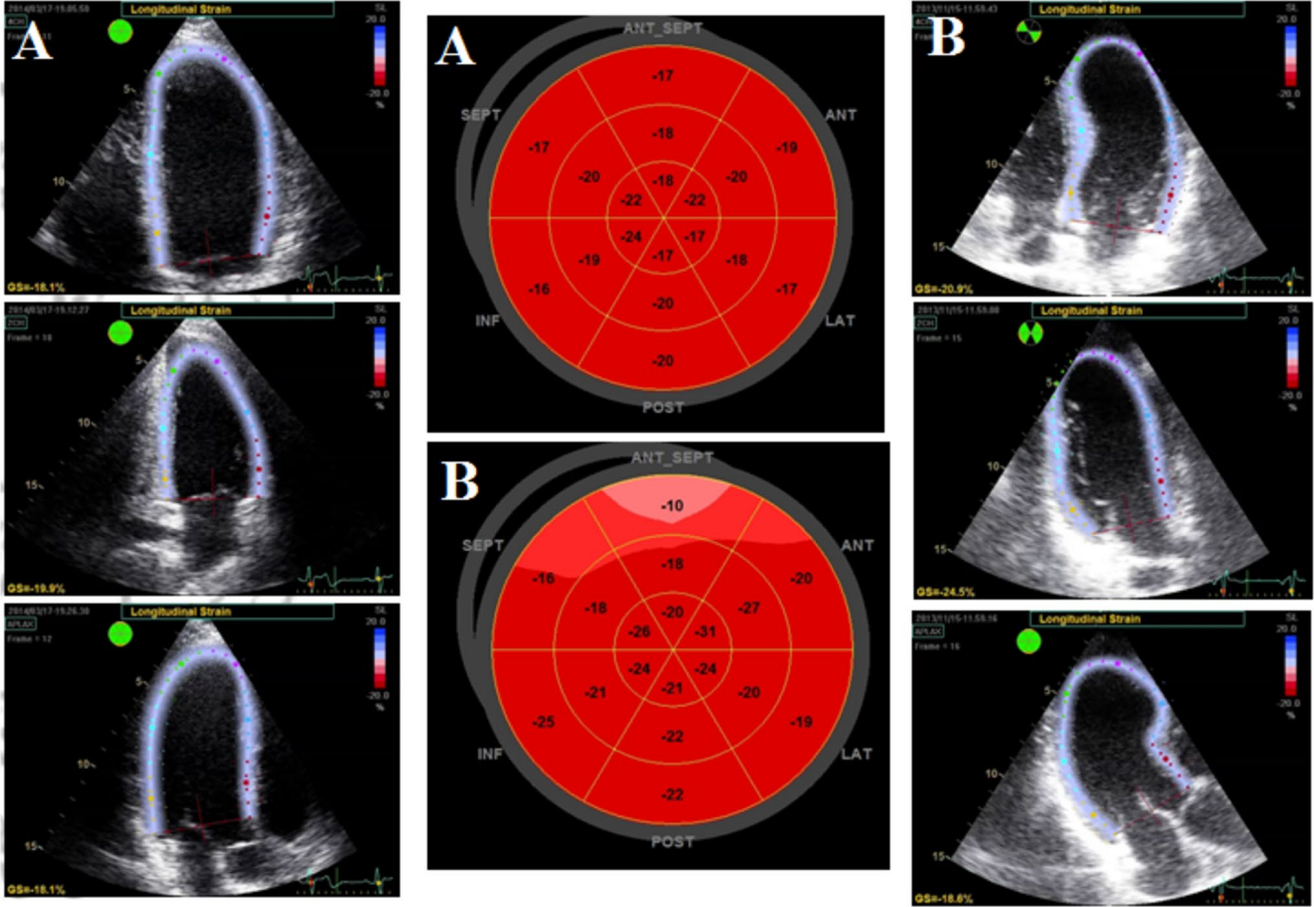
The right and left panels demonstrate the 3 apical views of LV coming from a normal athlete with a wall thickness of 13 mm (**A**) and from an athlete with genetic proof of hypertrophic cardiomyopathy and the same hypertrophy magnitude (**B**). The mid panel shows the bull’s eyes representing in color the segmental longitudinal strain values from the athlete (**A**) and HCM athlete (**B**). Impairment of basal septal segments (lower absolute strain values and different color) is evident in bull’s eye B compared to A, highlighting the potentials of longitudinal strain in the differentiation of the athlete’s heart from hypertrophic cardiomyopathy in the gray zone

Currently, the most commonly used imaging modality for the evaluation of myocardial deformation is speckle tracking echocardiography (STE). This technique allows the assessment of myocardial fibers’ strain and provides quantitative measurements of the regional and global LV systolic function. The term strain (“*ɛ*”) describes the fractional change in length, width, and/or thickness of a myocardial segment and is expressed as a percentage of the change in these physical dimensions during contraction. It can, thus, be categorized into longitudinal, circumferential, and radial strain. The rotational and twisting capacity of the LV can be assessed as well [11]. Patients with HCM are found to have lower Global Longitudinal Strain (GLS) compared to athletes (Fig. 1). These patients also have lower LV radial strain values but increased circumferential strain compared to controls [11]. In a study among football players, patients with HCM and controls, athletes showed higher values of left ventricular twist and torsion compared to patients [12]. This different response of myocardial strain to AH and HCM, therefore, opens new avenues of differential diagnosis and classification. Furthermore, a newer marker, Myocardial Work, was found reduced in patients with non-obstructive HCM. It correlates with maximal LV wall thickness and correspondingly worse long-term outcomes and may also contribute to differential diagnosis from AH in the gray zone [13]. Another index that may help is the assessment of strain after exercise. It has been suggested that exercise produces enhanced longitudinal strain in the middle to apical segments of the heart, representing some kind of functional reserve. Exercise produces only a mild increase in longitudinal strain in patients with HCM but not in twisting strain, whereas both parameters show an increase in healthy athletes [11].

#### Cardiac magnetic resonance (CMR)

It has been shown that the use of CMR can be a valuable tool to detect focal hypertrophic lesions located at the left anterior–lateral ventricular free wall and at the apex of the heart, that the ultrasound could not locate, leading thus to “missed” HCM cases [14]. Furthermore, CMR is able to more accurately quantify regions’ thickness that otherwise would fall into the gray zone if they were measured only by ultrasound [14]. CMR has also been utilized to evaluate negative T-waves found in athletes. In these cases, CMR is far more useful to diagnose athletes with cardiomyopathy compared to echocardiography alone [15]. It is worth mentioning that in healthy athletes the LV thickness/LV diastolic volume ratio has been found smaller than 0.15, whereas a greater value strongly supports the diagnosis of HCM. Moreover, the maximum/minimum diastolic thickness ratio in healthy athletes is lower than a value of 1.3 and greater in cases of pathological hypertrophy [16].

As far as the study of Late Gadolinium Enhancement (LGE) is concerned, in HCM, patchy, abnormal LGE segments are found in the most hypertrophic heart regions (which most commonly appear to be the anterior free wall and the basal anterior ventricular septum of the LV), with greater enhancement in patients that have diffuse rather than focal hypertrophy [17]. On the contrary, in athletes, fibrosis is mostly found at the insertion points [18]. Fibrosis in athletes is usually found in those with chronic involvement in high endurance sports [19].

In borderline cases of hypertrophy, the techniques of T1 mapping and Extracellular Volume (ECV) can prove to be of substantial use. In athlete’s heart, these two values appear lower, whereas they are higher in patients with HCM. Values of T1 > 1217 and ECV > 22.5% can very accurately (high specificity and sensitivity) distinguish patients with HCM from athletes with hypertrophy which falls in the range of the gray zone [20]. This ability stems from the fact that in athletes, the hypertrophy is due to an increase of the cellular component compared to the extracellular volume and thus leading to lower T1 values, while the opposite occurs in HCM where the presence of interstitial fibrosis results in higher T1 values [21]. Additionally, HCM patients demonstrate higher T2 values compared to athletes, even though T2 mapping may be affected by other factors as well (e.g., age and comorbidities) [22]. Apart from sarcomeric HCM, T1 and T2 mapping may also empower differential diagnosis of AH from other HCM phenocopies, including but not limited to cardiac amyloidosis or other infiltrative disorders as well as storage/metabolic diseases (Fabry, Danon’s etc.) which at their initial stages of concentric hypertrophy may impose difficult to solve diagnostic dilemmas [17–22].

**Fig. 2:**
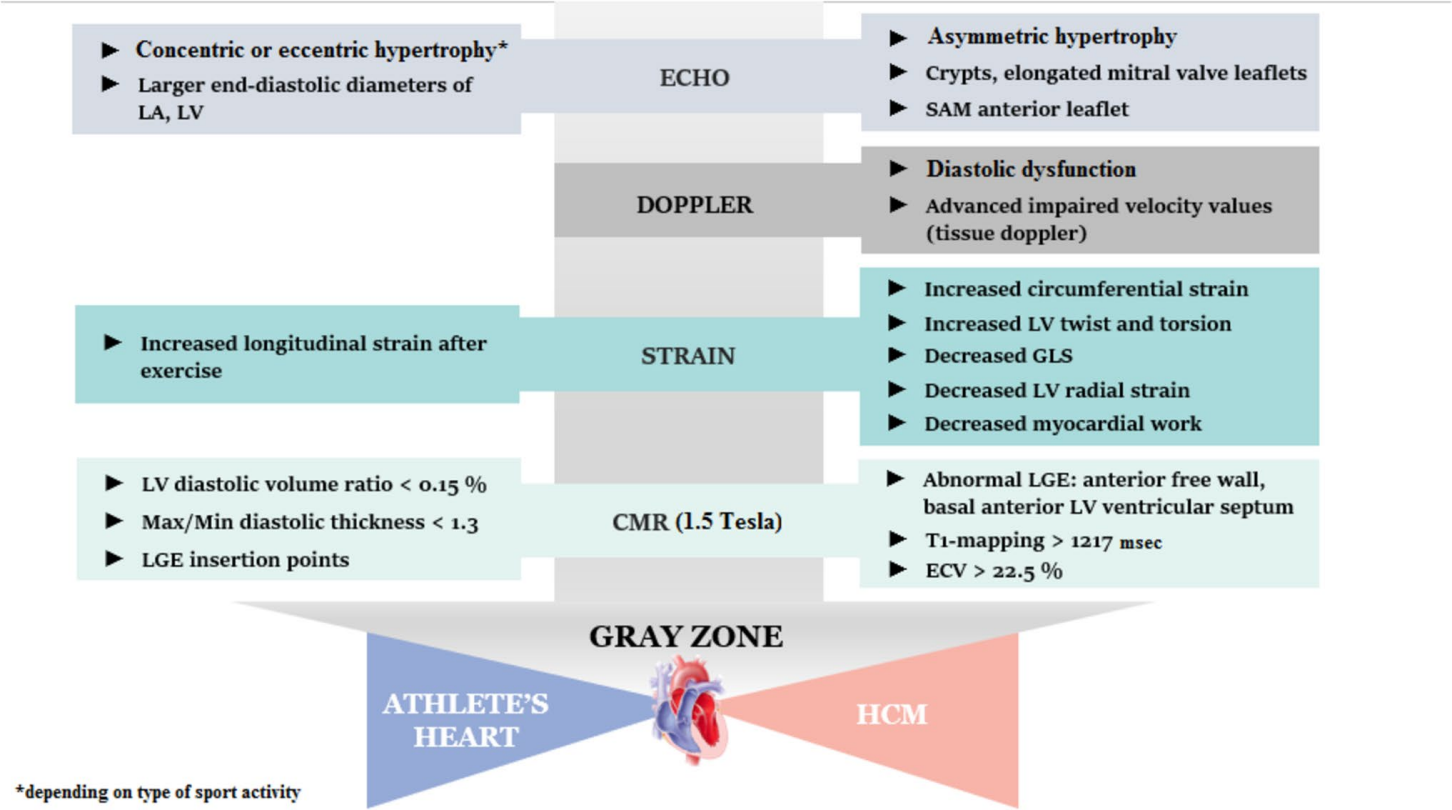
A schematic step-by-step algorithm presenting significant parameters from different imaging modalities that could be used to differentiate between athlete’s heart and hypertrophic cardiomyopathy in the gray zone

A new technique that aspires to reinforce the differentiation of the two entities discussed with the use of CMR is the “geometrical” analysis of the trabeculae of the LV, which is called Fractal Analysis. This method tries to quantify complex geometrical patterns in biological structures (in this case the LV trabeculae) with the aid of specialized software. This software uses the CMR images and converts them into a countable parameter, the Fractal Dimension (FD) [23]. A study that compared the FD values of the left ventricular trabeculae of athletes to those of patients with HCM found that the FD value of the latter is significantly greater than the former, displaying the potential of this technique [23]. A step-by-step approach in differentiating AH from HCM, using echo or CMR parameters is demonstrated in Fig. 2.

### Dilated cardiomyopathy (DCM)

#### Definition of gray zone

In athletes, LV end-diastolic diameter can exceed the limits set for the DCM diagnosis (> 58 mm in males and > 52 mm in females), as studies have shown that in male athletes it can reach even 66–70 mm while in female athletes 60–66 mm [7, 16]. Furthermore, in one out of six endurance athletes, a reduced LVEF < 50% and/or RVEF < 45% has also been noted [24]. These factors constitute a “gray zone” that has to be explored.

#### 2D speckle tracking echocardiography

In patients with DCM, reduced longitudinal strain (GLS) has been observed despite maintaining ejection fraction at normal values, highlighting the potential use of GLS as an early indicator in patients with DCM and, more broadly, in assessing systolic function [11]. Additionally, some data suggest that measuring GLS after exercise stimulus is useful for differentiating between DCM and AH, since an increase in total strain > 2% is in favor of athletic heart [25].

#### Stress echocardiography

Dobutamine stress echocardiography (DSE) is useful to assess myocardial contractile reserve, the presence of inducible ischemia, as well as evaluating the Coronary Flow Reserve (CFR). An increase in LVEF > 15% or in Wall Motion Score Index (WMSI) > 0.44 is indicative of maintenance of normal contractile reserve in athletes. In contrast, a biphasic response at least in two segments of the ventricular wall and/or an extended ischemic response during high dobutamine dose (or exercise) may help to identify ischemic cardiomyopathy. In idiopathic DCM, prolonged improvement in left ventricular function is observed, whereas the absence of an inotropic response identifies patients with severe cardiomyopathy [26, 27].

#### Cardiac magnetic resonance

Differentiating between AH and DCM is associated with some difficulties, especially in endurance athletes. The detection of LGE at the median wall of the LV can be pathognomonic for DCM. However, the lack of such a finding does not guarantee the elimination of DCM diagnosis [17]. The importance of LGE has been evidenced through studies that compared healthy athletes to patients with DCM. Researchers detected mid-wall and/or subendocardial LGE in the latter cases, which was not found in healthy individuals (without taking into account LGE at the insertion points) [28]. Also taking into consideration that past myocarditis may lead to mid-myocardial or sub-epicardial scar formation, detection of such fibrosis patterns may establish a causative relation in athletes presenting a DCM phenotype, facilitating thus differential diagnosis and preventing sudden cardiac death occurrence [29].

Studies of Exercise–CMR images (ex-CMR) attempted to bridge the gap in the diagnosis. Particularly, in one study, it was found that there are two parameters showing an increase during exercise in athletes, which was not observed in DCM patients. These parameters were (1) LVEF and (2) the Left Ventricular End-Systolic Pressure/Volume Ratio (LVESPVR). An increase of 11.2% in LVEF and 1.8 in LVESPVR during peak exercise compared to resting values was found to be of statistical importance and suggestive of AH [30], indicating that ex–CMR can be a useful differentiation tool [31].

The techniques of T1-mapping and ECV are also helpful. Increased values of both parameters in patients with DCM have been found to have a prognostic significance [32]. In athletes, the values of T1-mapping and ECV seem to be normal or even reduced [16].

T2-mapping could also play a role in the differential diagnosis, since it is a sensitive technique to detect oedema and inflammation. Higher values in patients with DCM are most likely related to the inflammatory nature of the cardiac remodeling, whereas this phenomenon is not observed during the normal, expected response of the heart to exercise [32].

Last but not least, studies of cardiac deformation (CMR-Feature Tracking), similar to those performed with the use of echocardiography, have shown that DCM and athlete’s heart are characterized by distinct profiles. Left ventricular Global Circumferential and Radial Strain (GCS and GRS accordingly) are the parameters with the highest sensitivity and specificity, while their values appear lower in pathological hearts [33].

**Fig. 3:**
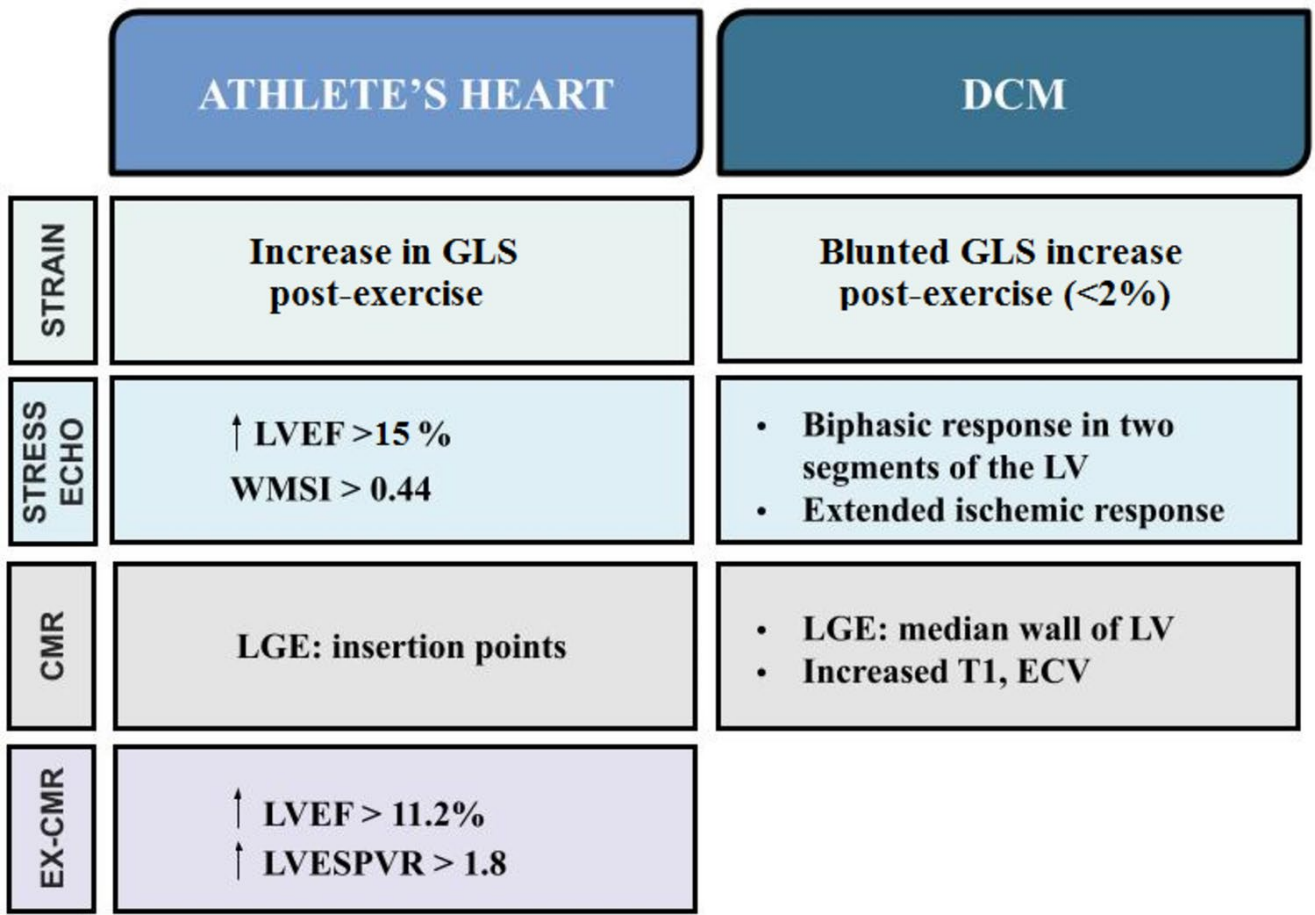
A schema demonstrating significant differences in imaging modalities’ parameters that could be used to differentiate between athlete’s heart and dilated cardiomyopathy in the gray zone. For terms’ definitions see inside text

A condensed guide to differentiate AH from DCM in the “gray zone” is demonstrated in Fig. 3.

### Arrhythmogenic right ventricular cardiomyopathy (ARVC)

#### Definition of gray zone

An enlargement of the RV is found both in athletes and in patients with ARVC, with athletes even fulfilling the ARVC dimensional criteria [34–37], leading once again to the formation of a “gray zone.” Taking also into consideration that athletes may frequently present premarure ventricular contractions originating from right ventricular outflow tract, or that they may demonstrate “marginal” LV systolic function along with ventricular ectopy, a situation resembling “Arrhythmogenic Cardiomyopathy (ACM)” [34–37], then the differential diagnosis between these two entities remains a significant clinical problem.

#### Echocardiography

The isolated dilatation of the right atrium is not sufficient evidence for the diagnosis of ARVC in an athlete, whereas if it is accompanied by RV systolic dysfunction, it can be a useful diagnostic marker for ARVC, especially among endurance athletes [38, 39]. Additionally, ARVC patients have also been found to have a greater indexed right/left atrial volumes ratio (RAVI/LAVI), with values > 1.11 being highly specific [40].

The maximum systolic strain of RV is significantly reduced in patients with ARVC compared to healthy controls [41]. Studies about RV strain in athletes are limited, while globally accepted normal values do not exist. A recent and comprehensive meta-analysis examined the structure and function of the right ventricle in ARVC and concluded that RV strain is significantly lower among these patients (range − 13% to − 21%) compared to controls (range − 27% to − 31%). Therefore, longitudinal RV strain values more positive than − 21% indicate pathology [41]. As a result, reduced RV strain can serve as an early indicator of the disease [42–44], something that is helpful in borderline cases of adolescent athletes as well [44].

In ALVC cases, patients can be distinguished via global LV systolic dysfunction (depression of LVEF or reduction of echocardiographic global longitudinal strain) and regional LV wall motion abnormalities (regional hypokinesia, akinesia, or dyskinesia), with or without LV dilatation [36, 45].

#### Cardiac magnetic resonance

Confirmation of ARVC diagnosis among athletes with borderline ultrasound findings necessitates the use of CMR [46]. The diagnosis of ARVC requires the presence of RV wall motion abnormalities or its asynchronous contraction [46]. Calculating the RV end-diastolic volume/LV end-diastolic volume ratio could be a helpful tool. Values lower than 1.2 are suggestive of AH [47]. As far as a functional impairment of RV is concerned, a decreased RVEF can be found in athletes, but this value is rarely lower than 45% [21, 47]. The detection of LGE in RV free wall [21] and/or in sub-endocardial or mid-wall regions of LV [47] confirms the diagnosis of ARVC [46]. The sensitivity of LGE, however, is higher when enhanced segments coincide with regions presenting motion abnormalities and/or fatty tissue infiltration [21, 46, 47].

**Fig. 4:**
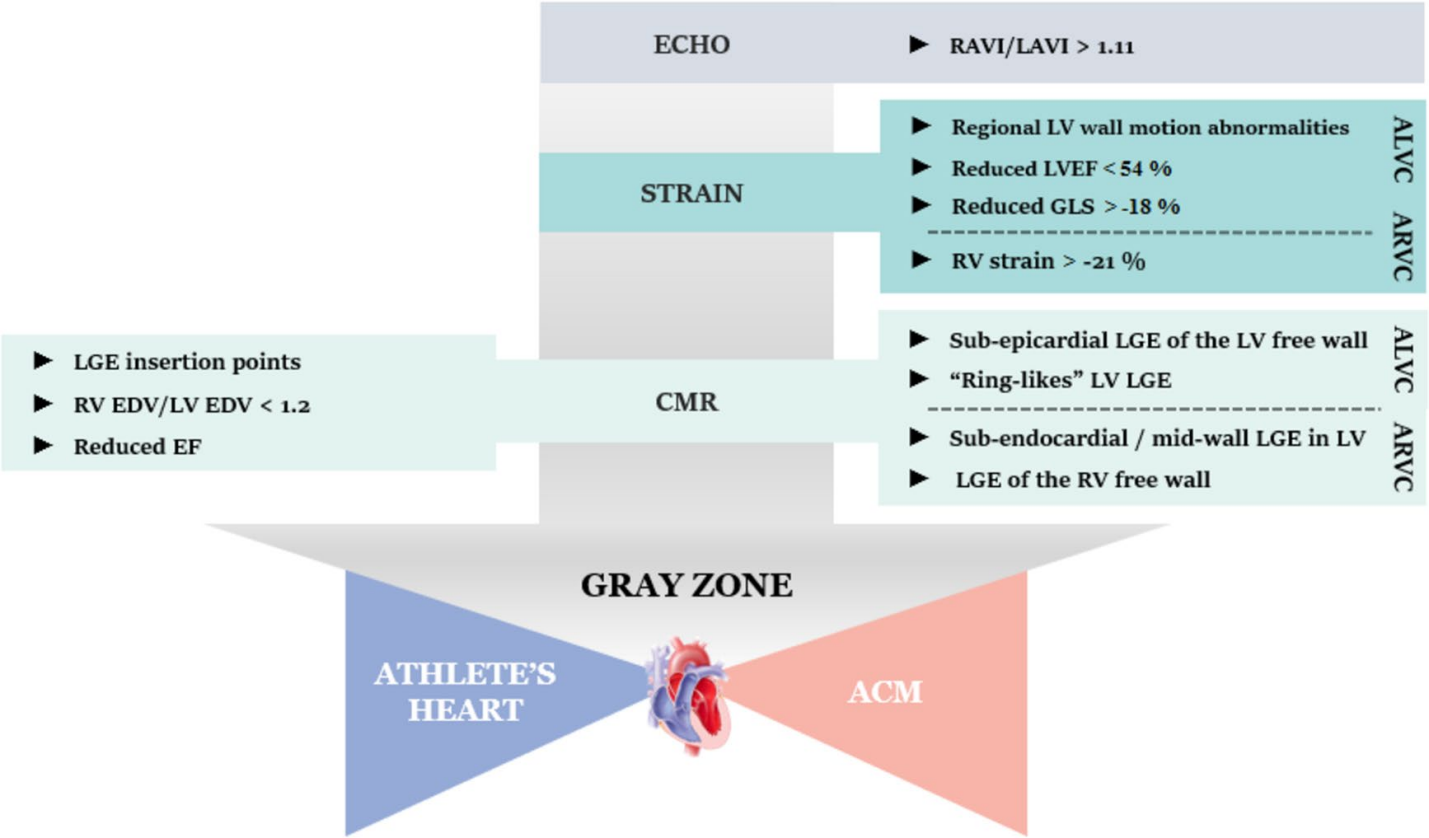
A schematic algorithm presenting significant parameters from different imaging modalities that could be used to differentiate between athlete’s heart and arrhythmogenic cardiomyopathy in the gray zone

Regarding ALVC, localization of LGE in a non-ischemic pattern is usually the rule among patients [46], with subepicardial layers of the LV free wall to be affected most [36, 46]. Non-ischemic LGE patterns are found in athletes as well, but these are located at the insertion points, with fibrotic tissue being also evident at lower and posterior LV wall [36, 46]. Novel studies suggests that “ring-like” or “Bull’s Eye” distribution of LV LGE is also suggestive of ACM [36], with desmoplakin and filamin-C genetic defects being mostly connected to such phenotype [36, 46]. Inconclusive CMR findings can be further elucidated by techniques of electroanatomical mapping (EAM) [36, 48]. A schematic algorithm for differentiating AH from ACM is shown in Fig. 4.

### Hypertrabeculated phenotype

#### Definition of gray zone

The term “hypertrabeculated phenotype” refers to the presence of a meshwork of numerous prominent muscle bands called trabeculations involving gaps in which there is blood flow, but no communication with the coronary circulation [49]. Hypertrabeculation is not a distinctive morpho-functional marker for cardiomyopathy, for which diagnosis relies on critical thinning of the compact layer with systolic dysfunction. Studies have documented that hypertrabeculation can appear as a phenotypic trait, particularly in athletes, as an adaptive response of myocardial architecture. This adaptation is believed to result from hemodynamic overload, suggesting that increased trabeculation allows for the same stroke volume to be generated with lower wall stress, and may occur irrespective of genetic susceptibility [50].

#### Echocardiography

Echocardiography is the main diagnostic tool. In particular, two-dimensional echocardiography is the most commonly used technique. Although there is no absolute agreement on the diagnostic criteria for cardiomyopathy with hypertrabeculated phenotype, two diagnostic models (Chin criteria, Jenni criteria) based mainly on left ventricular hypertrabeculation in echocardiography have been proposed [49]. In the assessment of myocardial work, a decrease in myocardial work index was observed. More specifically, both Global Constructive Work (GCW) and Myocardial Work Efficiency (MWE) are significantly lower in cardiomyopathy patients with hypertrabeculated phenotype and even marginally impaired ejection fraction, compared with healthy controls, a phenomenon that could be applied to the differential diagnosis of AH [49].

### Cardiac magnetic resonance

The diagnosis of hypertrabeculated phenotype with the use of CMR requires a non-compact/compact myocardium ratio during diastole greater than 2.3 [49, 50] while the trabeculated LV mass being 20% or more of the total cardiac mass has also been suggested as a diagnostic criterion [49, 50]. The most commonly affected regions are the median-upper parts of the posterolateral wall and the interventricular septum [21]. It is difficult to differentiate between AH and cardiomyopathy with hypertrabeculated phenotype. Firstly, because the above-mentioned criteria have a limited sensitivity, and secondly, myocardial hypertrabeculation has been found in athletes as well [49], most commonly in endurance athletes and athletes of African or Caribbean descent [16]. Useful findings are the higher T1-mapping values and the presence of LGE, which appears with a linear pattern located at the median cardiac layers without a definite relation to the non-compact areas [21].

## Conclusion

Almost 2% of AH phenotypes, either due to hypertrophy and/or dilatation developed, may overlap with various cardiomyopathies. Identification of the underlying cardiac condition is essential to reduce the possibility of sudden cardiac death during exercise and secure a safe involvement in training protocols. Echocardiography is the most widely available imaging method for assessing cardiac structure and function in athletes and is a first line tool not only for setting diagnostic dilemmas but also for resolving them. On the other hand, CMR provides a comprehensive evaluation of cardiac morphology and function, giving also the opportunity of tissue characterization. A combination of novel echo techniques, such as strain or myocardial work, and CMR imaging both at rest and after exercise may further enhance current diagnostic efforts and allow early identification even of mild pathologic phenotypes.

## Data Availability

No datasets were generated or analysed during the current study.

## References

[1] Carbone A, D’Andrea (2023) Cardiac dysfunction and athlete’s heart: new insights into pathophysiology and treatment, European Society of Cardiology. Available at: https://www.escardio.org/Journals/E-Journal-of-Cardiology-Practice/Volume-14/Cardiac-dysfunction-and-athlete-s-heart-new-insights-into-pathophysiology-and-treatment (Accessed: 16 May 2023).

[2] Martinez, M.W., Kim, J.H., Shah, A.B., Phelan, D., Emery, M.S., Wasfy, M.M., Fernandez, A.B., Bunch, T.J., Dean, P., Danielian, A., Krishnan, S., Baggish, A.L., Eijsvogels, T.M.H., Chung, E.H. and Levine, B.D. (2021). Exercise-induced cardiovascular adaptations and approach to exercise and cardiovascular disease: JACC state-of-the-art review. Journal of the American College of Cardiology, [online] 78(14):1453–1470. 10.1016/j.jacc.2021.08.003. 10.1016/j.jacc.2021.08.003 34593128

[3] Pelliccia A, Caselli S, Sharma S, Basso C, Bax JJ, Corrado D, D’Andrea A, D’Ascenzi F, Di Paolo FM, Edvardsen T, Gati S, Galderisi M, Heidbuchel H, Nchimi A, Nieman K, Papadakis M, Pisicchio C, Schmied C, Popescu BA, Habib G (2017) European Association of Preventive Cardiology (EAPC) and European Association of Cardiovascular Imaging (EACVI) joint position statement: recommendations for the indication and interpretation of cardiovascular imaging in the evaluation of the athlete’s heart. Eur Heart J 39(21):1949–1969. 10.1093/eurheartj/ehx532 10.1093/eurheartj/ehx532 29029207

[4] Beaudry R, Haykowsky MJ, Baggish A, La Gerche A (2016) A modern definition of the athlete’s heart—for research and the clinic. Cardiol Clin 34(4):507–514. 10.1016/j.ccl.2016.06.001 27692220 10.1016/j.ccl.2016.06.001

[5] D’Ascenzi, F., Anselmi, F., Piu, P., Fiorentini, C., Carbone, S.F., Volterrani, L., Focardi, M., Bonifazi, M. and Mondillo, S. (2019). Cardiac magnetic resonance normal reference values of biventricular size and function in male athlete’s heart. *JACC: Cardiovascular Imaging*, [online] 12(9), pp.1755–1765. 10.1016/j.jcmg.2018.09.021. 10.1016/j.jcmg.2018.09.021 30553678

[6] Albaeni A, Davis JW, Ahmad M (2021) Echocardiographic evaluation of the athlete’s heart. Echocardiography 38(6):1002–1016. 10.1111/echo.15066 33971043 10.1111/echo.15066

[7] Arbelo, E., Protonotarios, A., Gimeno, J.R., Arbustini, E., Barriales-Villa, R., Basso, C., Bezzina, C.R., Biagini, E., Blom, N.A., de Boer, R.A., De Winter, T., Elliott, P.M., Flather, M., Garcia-Pavia, P., Haugaa, K.H., Ingles, J., Jurcut, R.O., Klaassen, S., Limongelli, G. and Loeys, B. (2023). 2023 ESC Guidelines for the management of cardiomyopathies. European Heart Journal, [online] 44(37), p.ehad194. 10.1093/eurheartj/ehad194. 10.1093/eurheartj/ehad194 37622657

[8] Caruso, M., Garg, L. and Martinez, M.W. (2020). Cardiac imaging in the athlete: shrinking the ‘gray zone’. Current Treatment Options in Cardiovascular Medicine, 22(2). Available at: 10.1007/s11936-020-0802-8. 10.1007/s11936-020-0802-8 32016641

[9] Pagourelias ED, Efthimiadis GK, Kouidi E, Zorou PG, Giannoglou G, Deligiannis A, Athyros VG, Karagiannis A, Geleris P (2012) Efficacy of various ‘classic’ echocardiographic and laboratory indices in distinguishing the ‘gray zone’ between athlete’s heart and hypertrophic cardiomyopathy: a pilot study. Echocardiography 30(2):131–139. 10.1111/echo.12014 23167844 10.1111/echo.12014

[10] Sheikh, N., Papadakis, M., Schnell, F., Panoulas, V., Malhotra, A., Wilson, M., Carré, F. and Sharma, S. (2015). Clinical profile of athletes with hypertrophic cardiomyopathy. Circulation: Cardiovascular Imaging, 8(7). 10.1161/circimaging.114.003454. 10.1161/CIRCIMAGING.114.003454 26198026

[11] Forsythe, L., George, K. and Oxborough, D. (2018). Speckle tracking echocardiography for the assessment of the athlete’s heart: is it ready for daily practice?. Current Treatment Options in Cardiovascular Medicine, 20(10). 10.1007/s11936-018-0677-0. 10.1007/s11936-018-0677-0 PMC6132779 30146663

[12] Richand V, Lafitte S, Reant P, Serri K, Lafitte M, Brette S, Kerouani A, Chalabi H, Dos Santos P, Douard H, Roudaut R (2007) An ultrasound speckle tracking (two-dimensional strain) analysis of myocardial deformation in professional soccer players compared with healthy subjects and hypertrophic cardiomyopathy. Am J Cardiol 100(1):128–132. 10.1016/j.amjcard.2007.02.063 17599454 10.1016/j.amjcard.2007.02.063

[13] Hiemstra YL, van der Bijl P, el Mahdiui M, Bax JJ, Delgado V, Marsan NA (2020) Myocardial work in nonobstructive hypertrophic cardiomyopathy: implications for outcome. J Am Soc Echocardiogr 33(10):1201–1208. 10.1016/j.echo.2020.05.010 32680744 10.1016/j.echo.2020.05.010

[14] Maron MS, Maron BJ (2015) Clinical impact of contemporary cardiovascular magnetic resonance imaging in hypertrophic cardiomyopathy. Circulation 132(4):292–298. 10.1161/circulationaha.114.014283 26216086 10.1161/CIRCULATIONAHA.114.014283

[15] Schnell F, Riding N, O’Hanlon R, Axel Lentz P, Donal E, Kervio G, Matelot D, Leurent G, Doutreleau S, Chevalier L, Guerard S, Wilson MG, Carré F.(2015). Recognition and significance of pathological T-wave inversions in athletes. Circulation. 131(2):165–73. 10.1161/CIRCULATIONAHA.114.011038. 10.1161/CIRCULATIONAHA.114.011038 25583053

[16] Abulí, M.P., La Garza, M.S.-D. and Sitges, M. (2020). Differentiating athlete’s heart from left ventricle cardiomyopathies. Journal of Cardiovascular Translational Research, 13(3):265–273. Available at: 10.1007/s12265-020-10021-8. 10.1007/s12265-020-10021-8 32410209

[17] Gati, S., Sharma, S. and Pennell, D. (2018). The role of cardiovascular magnetic resonance imaging in the assessment of highly trained athletes. JACC: Cardiovascular Imaging, 11(2):247–259. 10.1016/j.jcmg.2017.11.016. 10.1016/j.jcmg.2017.11.016 29413645

[18] Małek ŁA, Bucciarelli-Ducci C (2020) Myocardial fibrosis in athletes-current perspective. Clin Cardiol. 10.1002/clc.23360 32189357 10.1002/clc.23360 PMC7403702

[19] van de Schoor, F.R., Aengevaeren, V.L., Hopman, M.T.E., Oxborough, D.L., George, K.P., Thompson, P.D. and Eijsvogels, T.M.H. (2016). Myocardial fibrosis in athletes. Mayo Clinic Proceedings, [online] 91(11):1617–1631. 10.1016/j.mayocp.2016.07.012. 10.1016/j.mayocp.2016.07.012 27720455

[20] Swoboda PP, McDiarmid AK, Erhayiem B, Broadbent DA, Dobson LE, Garg P, Ferguson C, Page SP, Greenwood JP, Plein S (2016) Assessing myocardial extracellular volume by T1 mapping to distinguish hypertrophic cardiomyopathy from athlete’s heart. J Am Coll Cardiol 67(18):2189–2190. 10.1016/j.jacc.2016.02.054 27151352 10.1016/j.jacc.2016.02.054

[21] Fogante, M., Agliata, G., Maria Sofia Basile, Paolo Compagnucci, Volpato, G., Falanga, U., Giulia Stronati, Guerra, F., Vignale, D., Esposito, A., Russo, A., Casella, M. and Salaffi, F. (2021). Cardiac imaging in athlete’s heart: the role of the radiologist. 57(5):455–455. 10.3390/medicina57050455. 10.3390/medicina57050455 PMC8148528 34066957

[22] Gastl M, Lachmann V, Christidi A et al (2020) Cardiac magnetic resonance T2 mapping and feature tracking in athlete’s heart and HCM. Eur Radiol 31(5):2768–2777. 10.1007/s00330-020-07289-4 33063183 10.1007/s00330-020-07289-4 PMC8043946

[23] Vilades D, Garcia-Moll X, Gomez-Llorente M et al (2021) Differentiation of athlete’s heart and hypertrophic cardiomyopathy by the fractal dimension of left ventricular trabeculae. Int J Cardiol 330:232–237. 10.1016/j.ijcard.2021.02.042 33621621 10.1016/j.ijcard.2021.02.042

[24] Claessen G, De Bosscher R, Janssens K, Young P, Dausin C, Claeys M, Claus P, Goetschalckx K, Bogaert J, Mitchell AM, Flannery MD, Elliott AD, Yu C, Ghekiere O, Robyns T, Sanders P, Kalman JM, Ohanian M, Soka M (2023) Reduced ejection fraction in elite endurance athletes: clinical and genetic overlap with dilated cardiomyopathy. PubMed. 10.1161/circulationaha.122.063777 10.1161/CIRCULATIONAHA.122.063777 PMC11062611 38109351

[25] Lancellotti, P., Pellikka, P.A., Budts, W., Chaudhry, F.A., Donal, E., Dulgheru, R., Edvardsen, T., Garbi, M., Ha, J.-W., Kane, G.C., Kreeger, J., Mertens, L., Pibarot, P., Picano, E., Ryan, T., Tsutsui, J.M. and Varga, A. (2016). The clinical use of stress echocardiography in non-ischaemic heart disease: recommendations from the European Association of Cardiovascular Imaging and the American Society of Echocardiography. Eur Heart J– Cardiovasc Imaging 17(11):1191–1229. 10.1093/ehjci/jew190. 10.1093/ehjci/jew190 27880640

[26] Pinamonti, B., Abate, E., De Luca, A., Finocchiaro, G. and Korcova, R. (2019). Role of cardiac imaging: echocardiography. Dilated Cardiomyopathy pp.83–111. 10.1007/978-3-030-13864-6_7. 32091726

[27] Palermi S , Cavarretta E, D’Ascenzi F, Castelletti S, Ricci F, Vecchiato M, Serio A, Cavigli L, Bossone E. (2023). Athlete’s heart: a cardiovascular step-by-step multimodality approach Rev. Cardiovasc. Med. 24(5): 151 10.31083/j.rcm2405151 10.31083/j.rcm2405151 PMC11273059 39076743

[28] Millar, L.M., Fanton, Z., Finocchiaro, G., Sanchez-Fernandez, G., Dhutia, H., Malhotra, A., Merghani, A., Papadakis, M., Behr, E.R., Bunce, N., Oxborough, D., Reed, M., O’Driscoll, J., Esteban, M.T.T., D’Silva, A., Carr-White, G., Webb, J., Sharma, R. and Sharma, S. (2020). Differentiation between athlete’s heart and dilated cardiomyopathy in athletic individuals. Heart. [online] 10.1136/heartjnl-2019-316147. 10.1136/heartjnl-2019-316147 32341137

[29] Javed W, Malhotra A, Swoboda P (2024) Cardiac magnetic resonance assessment of athletic myocardial fibrosis; benign bystander or malignant marker? Int J Cardiol 394:131382. 10.1016/j.ijcard.2023.131382 37741350 10.1016/j.ijcard.2023.131382

[30] Guido C, Schnell, F., Bogaert, J., Claeys, M., Pattyn, N., Frederik De Buck, Dymarkowski, S., Claus, P., Carré F, Van Cleemput J, La Gerche A and Heidbuchel H. (2018). Exercise cardiac magnetic resonance to differentiate athlete’s heart from structural heart disease. 19(9):1062–1070. 10.1093/ehjci/jey050. 10.1093/ehjci/jey050 29590340

[31] Le TT, Bryant JA, Ang BWY, Pua CJ, Su B, Ho PY, Lim S, Huang W, Lee PT, Tang HC, Chin CT, Tan BY, Cook SA, Chin CW (2020) The application of exercise stress cardiovascular magnetic resonance in patients with suspected dilated cardiomyopathy. J Cardiovasc Magn Reson 22(1):10. 10.1186/s12968-020-0598-4 32008575 10.1186/s12968-020-0598-4 PMC6996168

[32] Mordi, I., Carrick, D., Bezerra, H. and Tzemos, N. (2015). *T*_1_ and *T*_2_ mapping for early diagnosis of dilated non-ischaemic cardiomyopathy in middle-aged patients and differentiation from normal physiological adaptation. European Heart Journal – Cardiovascular Imaging, 17(7):797–803. 10.1093/ehjci/jev216. 10.1093/ehjci/jev216 26358692

[33] Małek ŁA, Mazurkiewicz Ł, Marszałek M et al (2021) Deformation parameters of the heart in endurance athletes and in patients with dilated cardiomyopathy—a cardiac magnetic resonance study. Diagnostics 11(2):374. 10.3390/diagnostics11020374 33671723 10.3390/diagnostics11020374 PMC7926616

[34] Coelho SA, Silva F, Silva J, António N (2019) Athletic training and arrhythmogenic right ventricular cardiomyopathy. Int J Sports Med 40(5):295–304. 10.1055/a-0750-5848 30865997 10.1055/a-0750-5848

[35] Zorzi A, Cipriani A, Mattesi G, Vio R, Bettella N, Corrado D (2020) Arrhythmogenic cardiomyopathy and sports activity. J Cardiovasc Transl Res 13(3):274–283. 10.1007/s12265-020-09995-2 32300932 10.1007/s12265-020-09995-2

[36] Corrado D, Anastasakis A, Basso C, Bauce B, Blomström-Lundqvist C, Bucciarelli-Ducci C, Cipriani A, De Asmundis C, Gandjbakhch E, Jiménez-Jáimez J, Kharlap M, McKenna WJ, Monserrat L, Moon J, Pantazis A, Pelliccia A, Perazzolo Marra M, Pillichou K, Schulz-Menger J, Jurcut R, Seferovic P, Sharma S, Tfelt-Hansen J, Thiene G, Wichter T, Wilde A, Zorzi A.(2024). Proposed diagnostic criteria for arrhythmogenic cardiomyopathy: European Task Force consensus report. Int J Cardiol. 395:131447. 10.1016/j.ijcard.2023.131447. 10.1016/j.ijcard.2023.131447 37844667

[37] Pagourelias, E.D., Kouidi, E., Efthimiadis, G.K., Deligiannis, A., Geleris, P. and Vassilikos, V. (2013). Right atrial and ventricular adaptations to training in male Caucasian athletes: an echocardiographic study. Journal of the American Society of Echocardiography, [online] 26(11):1344–1352. 10.1016/j.echo.2013.07.019. 10.1016/j.echo.2013.07.019 23978677

[38] La Gerche A, Burns AT, Mooney DJ, Inder WJ, Taylor AJ, Bogaert J, MacIsaac AI, Heidbüchel H, Prior DL (2011) Exercise-induced right ventricular dysfunction and structural remodelling in endurance athletes. Eur Heart J 33(8):998–1006. 10.1093/eurheartj/ehr397 22160404 10.1093/eurheartj/ehr397

[39] ‌Prior, D. and La Gerche, A. (2019) Exercise and arrhythmogenic right ventricular cardiomyopathy. Heart Lung Circ 29(4):547–555. 10.1016/j.hlc.2019.12.007 31964580 10.1016/j.hlc.2019.12.007

[40] Rossi VA, Niederseer D, Sokolska JM, Kovacs B, Costa S, Gasperetti A, Brunckhorst C, Akdis D, Tanner FC, Duru F, Schmied CM, Saguner AM (2021) A novel diagnostic score integrating atrial dimensions to differentiate between the athlete’s heart and arrhythmogenic right ventricular cardiomyopathy. J Clin Med 10(18):4094–4094. 10.3390/jcm10184094 34575205 10.3390/jcm10184094 PMC8472715

[41] Qasem, M., Utomi, V., George, K., Somauroo, J., Zaidi, A., Forsythe, L., Bhattacharrya, S., Lloyd, G., Rana, B., Ring, L., Robinson, S., Senior, R., Sheikh, N., Sitali, M., Sandoval, J., Steeds, R., Stout, M., Willis, J. and Oxborough, D. (2016). A meta-analysis for the echocardiographic assessment of right ventricular structure and function in ARVC: a Study by the Research and Audit Committee of the British Society of Echocardiography. Echo Research and Practice, [online] 3(3):95–104. 10.1530/ERP-16-0028. 10.1530/ERP-16-0028 PMC5076568 27686556

[42] Smiseth OA, Torp H, Opdahl A, Haugaa KH, Urheim S (2015) Myocardial strain imaging: how useful is it in clinical decision making? Eur Heart J 37(15):1196–1207. 10.1093/eurheartj/ehv529 26508168 10.1093/eurheartj/ehv529 PMC4830908

[43] Saberniak J, Hasselberg NE, Borgquist R, Platonov PG, Sarvari SI, Smith HJ, Ribe M, Holst AG, Edvardsen T, Haugaa KH (2014) Vigorous physical activity impairs myocardial function in patients with arrhythmogenic right ventricular cardiomyopathy and in mutation positive family members. Eur J Heart Fail 16(12):1337–1344. 10.1002/ejhf.181 25319773 10.1002/ejhf.181 PMC4278531

[44] Dorobantu DM, Riding N, McClean G, de la Garza MS, Abuli-Lluch M, Sharma C, Duarte N, Adamuz MC, Watt V, Hamilton RM, Ryding D, Perry D, McNally S, Stuart AG, Sitges M, Oxborough DL, Wilson M, Friedberg MK, Williams CA, Pieles GE (2023) The use of 2-D speckle tracking echocardiography in differentiating healthy adolescent athletes with right ventricular outflow tract dilation from patients with arrhythmogenic cardiomyopathy. Int J Cardiol 382:98–105. 10.1016/j.ijcard.2023.04.001 37030404 10.1016/j.ijcard.2023.04.001

[45] de la Guía-Galipienso F, Ugedo-Alzaga K, Grazioli G, Quesada-Ocete FJ, Feliu-Rey E, Perez MV, Quesada-Dorador A, Sanchis-Gomar F (2023) Arrhythmogenic cardiomyopathy and athletes: a dangerous relationship. Curr Probl Cardiol 48(9):101799. 10.1016/j.cpcardiol.2023.101799 37172878 10.1016/j.cpcardiol.2023.101799

[46] D’Ascenzi, F., Solari, M., Corrado, D., Zorzi, A. and Mondillo, S. (2018). Diagnostic differentiation between arrhythmogenic cardiomyopathy and athlete’s heart by using imaging. JACC: Cardiovascular Imaging, [online] 11(9):1327–1339. 10.1016/j.jcmg.2018.04.031. 10.1016/j.jcmg.2018.04.031 30190032

[47] Moccia E, Papatheodorou E, Miles CJ, Merghani A, Malhotra A, Dhutia H, Bastiaenen R, Sheikh N, Zaidi A, Sanna GD, Homfray T, Bunce N, Anderson LJ, Tome M, Behr E, Moon J, Sharma S, Finocchiaro G, Papadakis M (2022) Arrhythmogenic cardiomyopathy and differential diagnosis with physiological right ventricular remodelling in athletes using cardiovascular magnetic resonance. Int J Cardiovasc Imaging 38(12):2723–2732. 10.1007/s10554-022-02684-y 36445664 10.1007/s10554-022-02684-y

[48] Dello Russo A, Compagnucci P, Zorzi A, Cavarretta E, Castelletti S, Contursi M, D’Aleo A, D’Ascenzi F, Mos L, Palmieri V, Patrizi G, Pelliccia A, Sarto P, Delise P, Zeppilli P, Romano S, Palamà Z, Sciarra L (2023) Electroanatomic mapping in athletes: why and when. An expert opinion paper from the Italian Society of Sports Cardiology. Int J Cardiol 383:166–174. 10.1016/j.ijcard.2023.05.013 37178805 10.1016/j.ijcard.2023.05.013

[49] Srivastava S, Yavari M, Al-Abcha A, Banga S, Abela G (2022) Ventricular non-compaction review. Heart Fail Rev 27(4):1063–1076. 10.1007/s10741-021-10128-3 34232438 10.1007/s10741-021-10128-3

[50] Pittorru R, De Lazzari M, Migliore F, Frasson E, Zorzi A, Cipriani A, Brunetti G, De Conti G, Motta R, Perazzolo Marra M, Corrado D.(2024). Left ventricular non-compaction: evolving concepts. J Clin Med. 3(19):5674. 10.3390/jcm13195674. 10.3390/jcm13195674 PMC11477328 39407735

